# A tetrameric peptide derived from bovine lactoferricin as a potential therapeutic tool for oral squamous cell carcinoma: A preclinical model

**DOI:** 10.1371/journal.pone.0174707

**Published:** 2017-03-30

**Authors:** Víctor Alfonso Solarte, Paulette Conget, Jean-Paul Vernot, Jaiver Eduardo Rosas, Zuly Jenny Rivera, Javier Eduardo García, Martha Ligia Arango-Rodríguez

**Affiliations:** 1 Cellular and Molecular Physiology Group, Biomedical Research Institute, Faculty of Medicine, Universidad Nacional de Colombia, Bogotá, Colombia; 2 Center for Regenerative Medicine, School of Medicine Clínica Alemana Universidad del Desarrollo, Lo Barnechea, Santiago, Chile; 3 Department of Pharmacy, Faculty of Sciences, Universidad Nacional de Colombia, Bogotá, Colombia; 4 Department of Chemistry, Faculty of Sciences, Universidad Nacional de Colombia, Bogotá, Colombia; 5 Banco Multitejidos y Centro de Terapias Avanzadas, Fundación Ofalmológica de Santander, Clínica Carlos Ardila Lulle (FOSCAL Internacional), Bucaramanga, Colombia; University of South Alabama Mitchell Cancer Institute, UNITED STATES

## Abstract

Oral squamous cell carcinoma is the fifth most common epithelial cancer in the world, and its current clinical treatment has both low efficiency and poor selectivity. Cationic amphipathic peptides have been proposed as new drugs for the treatment of different types of cancer. The main goal of the present work was to determine the potential of LfcinB(20–25)_4_, a tetrameric peptide based on the core sequence RRWQWR of bovine lactoferricin LfcinB(20–25), for the treatment of OSCC. In brief, OSCC was induced in the buccal pouch of hamsters by applying 7,12-Dimethylbenz(a)anthracene, and tumors were treated with one of the following peptides: LfcinB(20–25)_4_, LfcinB(20–25), or vehicle (control). Lesions were macroscopically evaluated every two days and both histological and serum IgG assessments were conducted after 5 weeks. The size of the tumors treated with LfcinB(20–25)_4_ and LfcinB(20–25) was smaller than that of the control group (46.16±4.41 and 33.92±2.74 mm^3^ versus 88.77±10.61 mm^3^, respectively). Also, LfcinB(20–25)_4_ caused acellularity in the parenchymal tumor compared with LfcinB(20–25) and vehicle treatments. Furthermore, our results demonstrated that both LfcinB(20–25)_4_ and LfcinB(20–25) induced higher degree of apoptosis relative to the untreated tumors (75–86% vs 8%, respectively). Moreover, although the lowest inflammatory response was achieved when LfcinB(20–25)_4_ was used, this peptide appeared to induce higher levels of IgG antibodies relative to the vehicle and LfcinB(20–25). In addition the cellular damage and selectivity of the LfcinB(20–25)_4_ peptide was evaluated *in vitro*. These assays showed that LfcinB(20–25)_4_ triggers a selective necrotic effect in the carcinoma cell line. Cumulatively, these data indicate that LfcinB(20–25)_4_ could be considered as a new therapeutic agent for the treatment of OSCC.

## Introduction

Oral squamous cell carcinoma (OSCC), one of the ten most common cancers in the world, has a delayed clinical detection and poor prognosis [1]. The treatment of this pathology frequently requires combined therapies: surgery as the primary modality, and radiotherapy and chemotherapy as adjuvant treatments [2]. However, there is limited success associated with the use of these therapies, and patients report a variety of severe side effects caused by the lack of drug specificity [3]. In the face of these facts, researchers have proposed alternative therapeutic approaches in order to improve the treatment of OSCC [4, 5]. For instance, cationic amphipathic peptides (CAPs) have shown to be a novel promising tool for treating different types of cancer. Several studies have described their selectivity and cytotoxic activity in a wide range of tumors using different *in vitro* and *in vivo* cancer models [6–10]. These studies indicate that CAPs are likely to trigger fewer side effects [11–16] as well as induce immunogenic cell death, which in turn prevents cancer recurrence [10, 17, 18] and therefore, represents a significant advance towards more efficient cancer treatments. Moreover, additional studies have revealed that the high content of cationic and hydrophobic amino acids in CAPs, such as arginine and tryptophan, are critical for their antitumoral selective activity [19]. Specifically, these peptides are prone to interaction with anionic cell membrane surfaces [20], which are characteristic of cancer cells: they have more anionic molecules on the surface than non-cancerous cells. In addition, such anionic molecules are associated with phosohatidylserine, lipoproteins O-glycocylated mucines and sialic acid [21–23], and allow the peptides to be selective [23]. Regarding CAPs, their anticancer mechanism of action is suggested to be the induction of cell necrosis (via cell membrane lysis) or cell apoptosis (via slight damage to the plasma membrane or the mitochondrial lytic effect), which depend on the peptide concentration [23, 24].

Bovine Lactoferricin (LfcinB) is one of the most studied CAPs. Different amphipathic peptides have been derived from its sequence and have shown high selective cytotoxicity against various types of cancer, such as hematological malignancies, melanomas, and carcinomas [7, 14, 15, 18, 25–27]. In particular, several studies have reported that the core sequence 20-RRWQWR-25 (LfcinB(20–25)) of LfcinB is the responsible motif for the selective cytotoxic activity of the peptide, due to the well-defined amphipathic structure that the peptide adopts [23, 28, 29].

We have recently demonstrated that peptides derived from LfcinB(20–25) exhibited a specific cytotoxic effect in tumorigenic cell lines. Specifically, the tetrameric peptide LfcinB(20–25)_4_, containing four molecules of the monomer LfcinB(20–25) [29], caused a more selective cytotoxic effect on OSCC cells by activating a necrotic process [7]. Based on these findings, the present work evaluated the antitumoral effect of LfcinB(20–25)_4_ on a preclinical OSCC model generated in Syrian golden hamsters, in order to establish its potential use as a tool for human OSCC treatment.

## Materials and methods

### Peptide synthesis, purity and characterization

The linear form of LfcinB (LfcinB25), the monomeric (LfcinB(20–25)) and the tetrameric (LfcinB(20–25)_4_) peptides (Table 1) were synthesized using the SPPS-Fmoc/tBu method, as previously reported [30]. The purity (>90%) and the molecular weight of the peptides were determined via RP-HPLC analysis and MS MALDI-TOF, respectively. Peptides used for *in vivo* experiments were dissolved in 0.9% sodium chloride solution at 100, 200, and 300 μg/mL, while peptides used for *in vitro* assays were dissolved in culture medium without fetal bovine serum (FBS), at a concentration of 100 μg/mL.

**Table 1:** Bovine Lactoferricin-derived peptides used in this study.

Peptide	Amino acid sequence ^1^	Charge
LfcinB25	F**K**C**RR**WQW**R**M**KK**LGAPSITCV**RR**AF	+8
LfcinB(20–25)	**RR**WQW**R**	+3
LfcinB(20–25)_4_	(**RR**WQW**R**)_4_-K_2_-(Ahx)_2_-C_2_	+12

^1.^ Positively charged amino acids are shown in bold.

### Animal housing and development of the OSCC-hamster model

Eight-week old Syrian golden hamsters (*Mesocricetus auratus*, Charles River, USA) were used for the experiments. The animals were housed 1 animals per cage and were kept at constant temperature and humidity, with a 12 hours dark/light cycle and unrestricted access to standard diet and sterilized water. Nine animals were used as control (vehicle, 0.9% sodium chloride solution) and 28 were assigned for peptide evaluation. The animals were anesthetized with 20 mg/Kg of Xylazine (llium) and 20 mg/Kg Ketamine (Centrovet) when required. All animal procedures were approved by the Ethics Committee of the School of Medicine Clínica Alemana, Universidad del Desarrollo (approval ID:2011–14).

OSCC was induced by inserting a N°4 camel-hair brush previously soaked in 0.5% 7,12-Dimethylbenz(a)anthracene (DMBA, Sigma) dissolved in mineral oil, into the right buccal pouch of the hamsters, with the bristles pressed against the inner buccal pouch [31]. This procedure was performed three times a week during 12 weeks. After this, all animals treated with DMBA developed tumor lesions that are characteristic of OSCC in the buccal pouch. Once the animals had developed OSCC, the peptides and vehicle were administered for 5 weeks without interrupting the application of DMBA. All animals were euthanized by an overdose of anesthesia (ketamine 50 mg/kg–xylazine 50 mg/kg, Centrovet) prior to collection and fixation of tissues.

Animals were monitored daily for changes in general health and signs of stress, including body weight loss, diarrhea, changes in food/water intake, appearance (hunched posture, sunken eye, labored breathing) and behavior (signs of lethargy and weakness). The hamsters were euthanized when the maximum tumour volume was reached (200 mm^3^) or if there was more than 20% weight loss.

### Macroscopic analysis of OSCC

After inducing OSCC for 12 weeks, the tumors in the buccal pouches were measured with a digital caliper (Mitutoyo) and photographed using a digital camera (FUJIFILM-Finepix HS20 EXR) every two days for five weeks. The tumor volume was calculated using the formula: *V* = 0.52 * (*length*) * (*width*)^2^ [32].

### Peptide administration

Once the lesions had reached the carcinoma stage, the animals were randomly distributed into three treatment groups: LfcinB(20–25)_4_, LfcinB(20–25), and vehicle. The hamsters were anesthetized, and their buccal pouches were exposed with surgical forceps. A 23-gauge needle (Terumo) was used to intratumorally inject 300 μL of either peptide, or the vehicle. LfcinB(20–25)_4_ was administered at 100 μg/mL, 200 μg/mL and 300 μg/mL, while LfcinB(20–25) was administered at 300 μg/mL. Also, the tumors were treated in two different ways: i) acute treatment: the peptides were delivered in 3 doses (n = 2 animals for each group), and ii) chronic treatment: the peptides were delivered in 15 doses (n = 5 animals for each peptide group, and n = 7 for the vehicle group).

### Microscopic analysis of OSCC

After five weeks of treatment, the hamsters were euthanized with an overdose of 20 mg/Kg Xylazine and 20 mg/Kg Ketamine by intraperitoneal injection.

Buccal pouches were procured, and tumors were resected for standard histological analyses. In brief, tumor specimens were fixed in 10% buffered formalin (Merck) and embedded in paraffin (Merck). Four μm sections were cut from each embedded sample, deparaffinized with Neoclear (Merck), and rehydrated with graded alcohols. Haematoxylin and Eosin stains (H&E) (Merck) were applied to the sections, which were imaged using a light microscope (DM2000, Leica) with a digital camera (DFC295, Leica). For OSCC assessment, the carcinoma stage was defined as previously described [25]. The histological evaluation was performed by two independent observers blinded to outcome.

### Apoptosis and necrosis assessment

Tissue sections of 4 μm were cut and deparaffinized from each embedded sample. Rehydrated sections were permeabilized (0.1% TBS-Tween, Sigma) prior to blocking with 5% FBS (Gibco). Primary antibody for human Cleaved-Caspase-3 (Cell Signaling) was diluted 1:200, applied to the sections and incubated overnight at 4°C. Bound primary antibody was detected by exposing the sections to Alexa Fluor 488 conjugate goat anti-rabbit (1:400 dilution) (Abcam) for 2 h at room temperature. Cross-reactivity of the secondary antibody was tested by incubating some samples without the primary antibody. Similarly, additional tissue sections were deparaffinized, rehydrated, and digested with 20 μg/mL proteinase K (Invitrogen). Terminal deoxynucleotidyl transferase dUTP nick 3’endlabeling (TUNEL) kit (Promega) was used to label the sections, following manufacturer instructions. Nuclei were counterstained with DAPI (1:1500 dilution) (Invitrogen). Stained sections were imaged using a light microscope (Leica DM2000) with a digital camera (Leica DFC 295) and 40X magnification. Images were analyzed with Image J software (NIH) in order to calculate the percentage of apoptosis and necrosis, expressed as the number of Cleaved Caspase-3 and TUNEL positive cells per 1000 cells, observed in ten representative optical sections (areas with extensive necrosis were avoided).

### Leukocyte infiltration assessment

Valid histological criteria in regular H&E staining was standardized by a qualified pathologist in order to assess the degree of leukocyte infiltration (unquantifiable subjective parameter). For this purpose, an estimation of the area occupied by inflammatory cells (lymphocytes, plasma cells, eosinophils, macrophages, neutrophils, among others) was determined in the tumor parenchyma. The following scale was used: absent (no apparent inflammatory response); mild (< 10% of the area covered by inflammatory cells); moderate (10 to 50% of the area covered by inflammatory cells) and severe (> 50% of the area covered by inflammatory cells).

The degree of leukocyte infiltration was labeled according to the following nomenclature: absent (-), mild (+), moderate (++) and severe (+++).

### IgG antibody quantification

IgG antibodies were quantified by ELISA following manufacturer’s instructions (Abcam). In brief, specific anti-peptide IgG amount was determined by coating MaxiSopr flat-bottom 96 well plate (Nunc) overnight at 4°C with the appropriate peptide (100 μg/mL) [33]. The coated wells were then rinsed with 0.1% PBS-Tween, blocked with 5% w/v nonfat dry milk (Nestle) and rinsed again with PBS and 0.1% PBS-Tween. After washing, serum samples were incubated following manufacturer’s instructions.

### Cell culture

The tongue squamous cell carcinoma line CAL27 (ATCC, CRL­2095) and the immortalized non-tumoral human epithelial esophagus cell line Het-1A (ATCC, CRL­2692) were purchased from ATCC (Manassas, VA). CAL27 cells were cultured in Dulbecco's Modified Eagle's Medium (DMEM; Gibco) supplemented with 10% fetal bovine serum (FBS; Gibco). Moreover, Het-1A cells were cultured in Bronchial Epithelial Cell Growth Medium (BEBM) without antibiotics, using the supplements recommended by Lonza/Clonetics Corporation. In addition, the Het-1A culture flasks were pre-coated with fibronectin (0.01 mg/mL), bovine collagen type I (0.03 mg/mL), and bovine serum albumin (0.01 mg/mL) by adding media containing these proteins and incubating the flasks for 24 h prior to use. All cells were maintained at 37°C and 5% CO_2_ humidified atmosphere. Furthermore, cell stocks were prepared, thawed periodically and used in early subcultures (not exceeding 5–10 population doublings). The viability of the obtained cells was estimated to be higher than 97% on the basis of Trypan blue exclusion.

### Transmission electron microscopy

Cells were seeded on 35 mm plates at a density of 3×10^5^ cells/well (either CAL27 or Het-1A) in order to achieve an initial cell confluence of approximately 70%. Following cell adhesion, the culture medium was removed and either peptides (LfcinB25, LfcinB(20–25), and LfcinB(20–25)_4_) or Triton X-100 (T-X100, 0.1% v/v, positive control) were added to each well at 100 μg/mL. After 15 or 45 min of incubation at 37°C, treated CAL27 and Het-1A cells were fixed with 2.5% glutaraldehyde (Merck) dissolved in 0.1 M sodium cacodylate buffer (pH 7.0, Sigma) for 30 min. Cells were then harvested using scrapers (2.4 mm thick), followed by centrifugation at 400 g for 10 min. The pellets were fixed overnight at room temperature, and washed 3 times with the cacodylate buffer for 2 h. After this, they were fixed with 1% aqueous osmium tetroxide (Sigma) for 90 min and washed with deionized water. Subsequently, samples were stained with 1% uranyl acetate (Sigma) for 60 min and then dehydrated in a series of graded acetone solutions (Sigma) for 20 min each (50, 70, 95, and 100%), after which they were immersed in acetone:EPON (Sigma) solution (1:1) overnight. Thereafter, the polymerization was conducted at 60°C for 48 h. Sixty nm thick sections were cut using an ultramicrotome (Sorvall MT5000) and stained with 4% uranyl acetate in methanol and lead citrate (Merck) for 2 and 5 min, respectively. Samples were examined using a transmission electron microscope (Tecnai 12 Philips) at 80 kV accelerating voltage.

### Statistical analysis

Data are reported as mean ± standard error of the mean. Comparison of experimental groups was performed using analysis of variance (ANOVA) followed by a Tukey's or Dunn’s multiple test (Stat Graph Prism 6.0 software), with p < 0.05 considered statistically significant.

## Results

### Antitumoral effect of LfcinB(20–25)_4_ peptide in the OSCC hamster model

**Fig 1:**
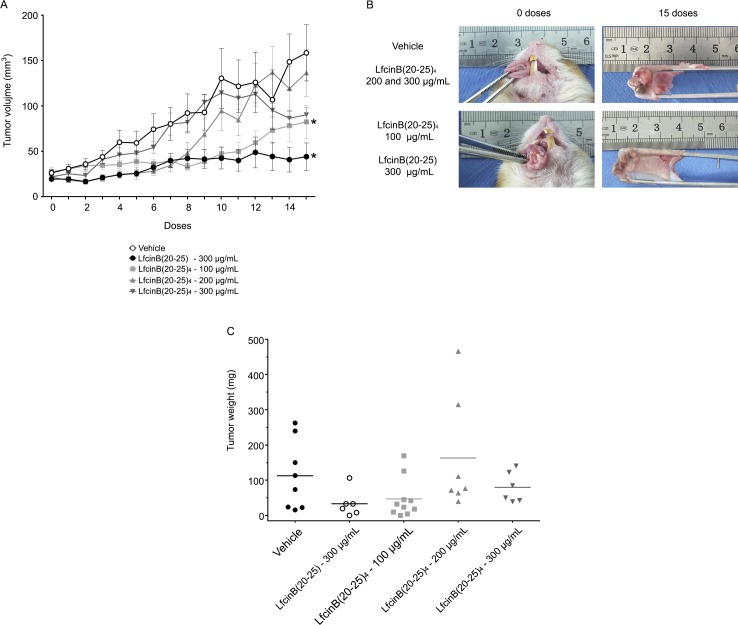
Antitumoral effect of LfcinB peptides in OSCC hamster model. Five weeks after administration of the peptides or the vehicle, macroscopic characteristics of the tumors were evaluated. **(A)** Quantitative analysis of tumor volume at different doses. **(B)** Representative macroscopic images of buccal pouch pre- and post-treatments. **(C)** Quantitative analysis of tumor weight. Data of n = 7 animals for vehicle group, n = 5 animals for peptide groups. ^*^indicates a significant difference (p < 0.05) relative to the peptide and vehicle treatments.

In order to study the antitumoral activity of the LfcinB(20–25)_4_ and LfcinB(20–25) peptides, the hamsters were chronically treated with each of them (Fig 1 and S1 Fig). The volume of carcinoma lesions was 23.41±1.55 mm^3^, which was measured before peptide administration (0 doses).

After 8 doses (2.5 weeks of treatment), the groups treated with either LfcinB(20–25)_4_ at 100 μg/mL and 200 μg/mL, or LfcinB(20–25) at 300 μg/mL had similar tumor sizes (32.81±3.84 mm^3^; 34.42±6.55 mm^3^ and 42.28±10.09 mm^3^, respectively), which were smaller than those of the groups treated with vehicle and LfcinB(20–25)_4_ at 300 μg/mL (80.19±18.33 mm^3^ and 81.92±11.63 mm^3^, respectively). Furthermore, after 15 doses (5 weeks of treatment), the groups treated with LfcinB(20–25)_4_ at 100 μg/mL and LfcinB(20–25) at 300 μg/mL had a significantly smaller tumor size (82.34±18.05 mm^3^ and 44.18±15.09 mm^3^, respectively) relative to the vehicle, LfcinB(20–25)_4_ at 200 μg/mL, and LfcinB(20–25)_4_ at 300 μg/mL groups (158.61±30.98 mm^3^; 136.59±26.12 and 90.32±8.35 mm^3^, respectively) (p<0.05, Fig 1A). Similar results were observed in the macroscopic images of the buccal pouches of the different groups, as depicted in Fig 1B and S1 Fig. In particular, buccal pouches for the groups treated with vehicle and LfcinB(20–25)_4_ at 200 and 300 μg/mL were reduced and stiff, showing traits of necrosis in the tumor. In contrast, the lesions of the groups treated with LfcinB(20–25)_4_ at 100 μg/mL and LfcinB(20–25) at 300 μg/mL did not extend into the connective tissue, and contained fewer necrotic traits. Also, these buccal pouches were the least reduced and stiff (Fig 1B). In addition to tumor volume, tumor weight was also assessed. Although the data were not statistically significant, the average tumor weight in LfcinB(20–25)_4_ at 100 μg/mL and LfcinB(20–25) at 300 μg/mL was lower compared to the vehicle, LfcinB(20–25)_4_ at 200 μg/mL and LfcinB(20–25)_4_ at 300 μg/mL groups (46.97±17.72 and 33.23±15.60 mg vs 112.61±34.56, 163.20±61.42, and 79.86±17.40 mg, respectively) (Fig 1C).

### Histological characteristics of OSCC tumors

**Fig 2:**
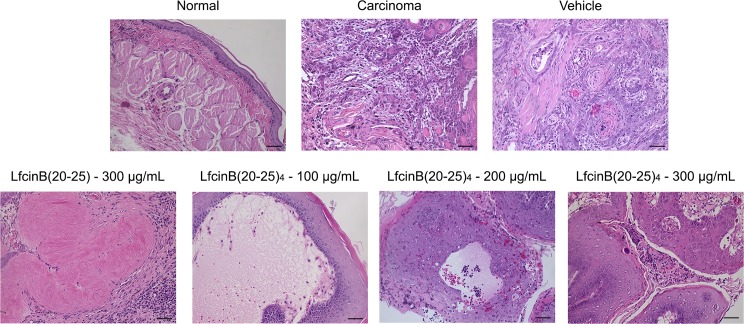
Histological characteristics of OSCC tumors. Following five weeks of treatment, the histological characteristics of OSCC tumors were evaluated**.** Images show sections of the oral buccal pouch stained with H&E, emphasizing the stroma area. Histological analysis showed acellularity in the stroma of animals treated with LfcinB(20–25)_4_ and LfcinB(20–25). Scale bar = 100 μm.

Following five weeks of treatment, histological characteristics of OSCC tumors were examined in each of the animal groups. Hamsters treated with vehicle developed tumors with histological features of the carcinoma stage, including abundant cell and nuclear pleomorphisms, loss of stratification, discontinuous basement membrane, stroma cell invasion and abundant inflammatory foci (Fig 2). Conversely, animals prescribed with LfcinB(20–25)_4_ at 100 μg/mL (3–5 animals) and 200 μg/mL (4–5 animals) exhibited partial acellularity in the stroma, feature that was not observed in the groups treated with LfcinB(20–25)_4_ and LfcinB(20–25) at 300 μg/mL (only 1 out of 5 animals showed this feature) (Fig 2).

### Apoptosis in OSCC tumors

**Fig 3:**
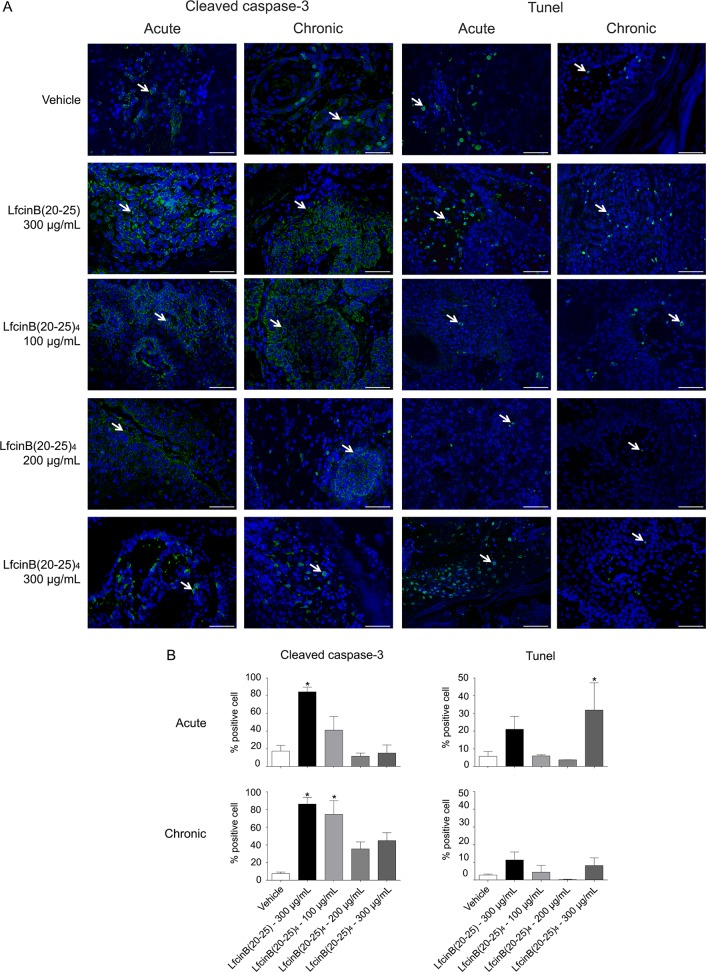
Detection and measurement of cell death in OSCC tumors. Cell apoptosis and necrosis were assessed after five weeks of vehicle or peptide administration. **(A)** Images of buccal pouch sections show Cleaved Caspasa-3 positive cells and TUNEL reactivity (green). Nuclei were stained with DAPI (blue). Scale bar = 50 μm. (**B)** Quantitative analysis of the Cleaved Caspasa-3 positive cells and TUNEL reactivity per 1000 cell nuclei in acute or chronic treatments. Representative data of 2 animals per group. ^*^indicates a significant difference (p < 0.05) relative to vehicle treatment.

The levels of Cleaved Caspase-3 and DNA breaks under acute and chronic treatment were evaluated. The percentage of apoptosis (Cleaved Caspase-3) was higher in animals treated with LfcinB(20–25)_4_ at 100 μg/mL and LfcinB(20–25) at 300 μg/mL compared with the vehicle group and animals treated with LfcinB(20–25)_4_ at 200 μg/mL or 300 μg/mL under acute treatment (47.05% and 85.5% vs. 14.80%; 13.45% and 13.55%, respectively). Similarly, the percentage of apoptosis was significantly higher in the LfcinB(20–25)_4_ at 100 μg/mL and LfcinB(20–25) at 300 μg/mL groups relative to LfcinB(20–25)_4_ at 200 μg/mL or 300 μg/mL groups under chronic treatment (82.85% and 84.25% vs. 7.30%; 37.25% and 45.95% respectively) (Fig 3A and 3B). On the other hand, in the acute treatment, hamsters treated with LfcinB(20–25)_4_ at 300 μg/mL showed the highest percentage of necrotic cells (DNA breaks) (31.90%) (Fig 3A and 3B).

### Leukocyte infiltration in OSCC tumors

**Fig 4:**
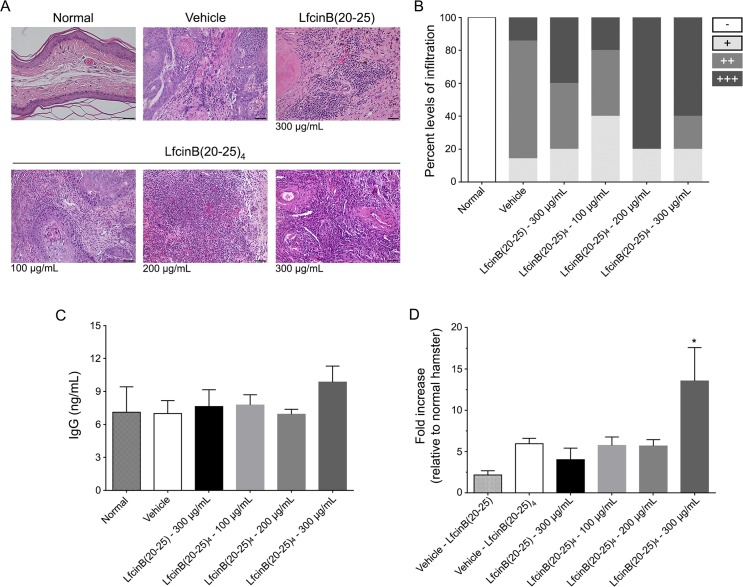
Degree of leukocyte infiltration in OSCC tumors and quantification of peptide-specific IgG. Following five weeks of treatment, tumor leukocyte infiltration and the presence of peptide-specific IgG were studied. **(A)** Sections of the oral buccal pouch stained with H&E, showing the inflammatory infiltrate. Scale bar = 100 μm. **(B)** Quantitative analysis of the leukocyte infiltrate score: absent (-), mild (+), moderate (++) and severe (+++). **(C)** Quantitative analysis of the amount of hamster IgG in serum of animals treated with peptides or vehicle. **(D)** Increase of the levels of peptide-specific hamster IgG relative to normal hamster IgG levels (absorbance at 450 nm). Representative data of 5 animals per group. ^*^indicates a significant difference (p < 0.05) relative to the peptide and vehicle treatments.

To determine the stimulation of any type of immune response caused by the peptide doses in the animals chronically treated, a histological study of the magnitude of leukocyte infiltration (lymphocytes, plasma cells, eosinophils, macrophages, neutrophils, among others) was performed. Higher leukocyte infiltration (score: +++) was found into the tumor parenchyma of animals treated with LfcinB(20–25)_4_ at 200 μg/mL and 300 μg/mL, relative to those treated with vehicle, LfcinB(20–25)_4_ at 100 μg/mL and LfcinB (20–25) at 300 μg/mL (80% and 60% versus 14%; 20% and 40%, respectively) (Fig 4A and 4B).

### Presence of peptide-specific IgG

In order to determine whether a humoral immune response was triggered, the levels of peptide-specific IgG were quantified. A mild increase in IgG production was found in the animals treated with LfcinB(20–25)_4_ at 300 μg/mL compared with the other groups (9.85 ±1.46 vs. 7.28±0.17 ng/mL) (Fig 4C). In addition, the levels of peptide-specific antibodies were significantly higher in the hamsters that received LfcinB(20–25)_4_ at 300 μg/mL compared with the other groups (13.53±4.05 vs. 4.71±0.72-fold increase in the absorbance, p<0.05) (Fig 4D).

### *In vitro* cytotoxic effect of LfcinB(20–25)_4_

**Fig 5:**
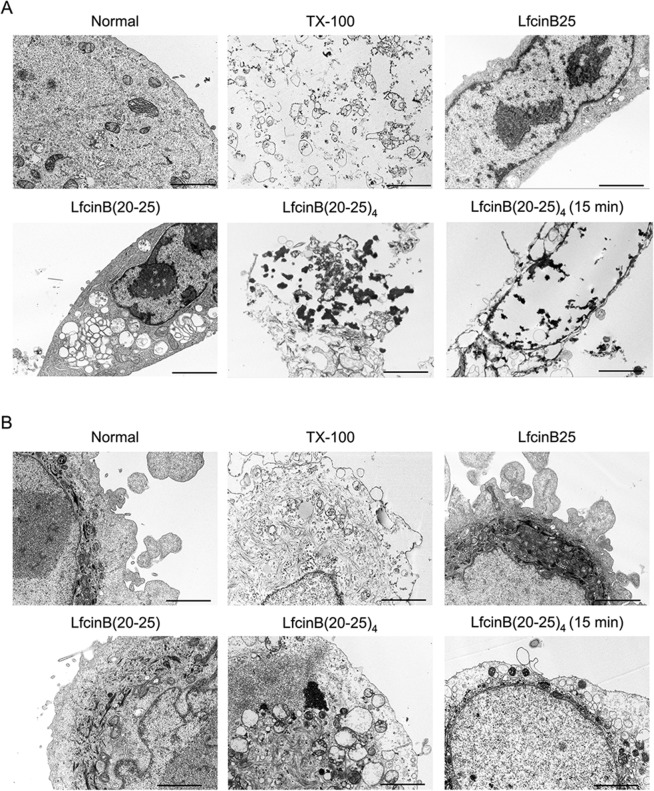
*In vitro* cytotoxic effect of LfcinB (20–25)_4_. Structural damage caused by LfcinB (20–25)_4_ in CAL27 and HET-1A cell lines was estimated via transmission electron microscope. Images show sections of **(A)** CAL27 and **(B)** HET-1A cells, pre- and post- treatments. Scale bar = 2 μm.

In order to estimate the extent of cellular harm and selectivity of LfcinB(20–25)_4_, the structural damage in the OSCC CAL27 and non-tumorigenic Het-1A cell lines was evaluated via transmission electron microscopy. The results revealed membranolytic activity (disruption of cell membrane, cytoplasm, and nuclear matrix) in CAL27 cells treated with LfcinB(20–25)_4_ at 100 μg/mL for 15 or 45 min, a response that was observed with the 0.1% T-X100 treatment. On the contrary, both LfcinB25 and LfcinB(20–25) at 100 μg/mL only induced cytoplasmic vacuole formation, in which LfcinB(20–25) treatment had not only the highest number of vacuoles but also the biggest size of vacuoles (Fig 5A and S2 Fig). Despite that, the Het-1A cells developed minor structural damage, and the amount of cytoplasmic vacuoles was lower for all peptides, relative to CAL27 cells. Nevertheless, LfcinB(20–25)_4_ treatment showed evident formation of cytoplasmic vacuoles (Fig 5B and S3 Fig).

## Discussion

The present study was conducted to examine the antitumor effect of LfcinB(20–25)_4_ on an OSCC golden Syrian hamster experimental model, which is recognized as the most characterized system to analyze OSCC. This model closely mimics not only the carcinogenic activation markers and oxidative stress but also the cell proliferation, apoptosis, invasion and angiogenesis of human OSCC. In addition, it is a system that allows to better understand the molecular mechanisms of neoplastic transformation as well as to probe the efficacy of new drugs. Furthermore, it mimics the grossly visible changes are grossly visible and patognomonic characteristics of each stage [34, 35].

Therapeutic agents can be administrated systemically or intratumorally. In the case of OSCC, intratumorally administration produces a superior antitumor effect with less severe side effects (mucosal damage, nausea, vomiting, anorexia, dysphagia, nephrotoxicity, ototoxicity, pain and hematologic toxicity). Nevertheless, when therapeutic drugs are locally administered, they need to be both intrinsically bioactive and soluble. Also, they should not require activation in other sites of the body and produce significant damage to normal tissues [36]. In fact, the used peptides in the present work meet all these requirements.

Our data revealed a significant antitumor effect associated with LfcinB(20–25)_4_ at the lowest dose (100 μg/mL or 24.25 μM) and LfcinB(20–25) at 300 μg/mL (304.21 μM). We also found that the tetrameric peptide did not show a dose-dependent concentration effect, a fact that could suggest that the peptide aggregated at higher concentrations, as reported previously by Farnaud et al. [37] and Chapple et al. [38]. Specifically, they report that human lactoferricin-derived amphipathic peptides, BLP-2 (RRWQWRMKKLG) and HLP-2 (FQWQRNMRKVR) tend to form beta sheet conformations that lead to the formation of aggregates due to electrostatic and hydrophobic interactions among their multiple chains. Thus, further studies should focused on exploring this hypothesis.

Furthermore, the effective molar concentration of the LfcinB(20–25)_4_ was 12.5 times lower than the LfcinB(20–25) one, suggesting that the tetrameric structure enhanced the cytotoxic effect. Similar results have been observed with dendrimeric peptides previously proposed as drug delivery systems, in which the dendrimeric structure increased the efficacy of the peptide at a lower molar concentration [39–41]. On the other hand, animals treated with LfcinB(20–25)_4_ at concentrations greater than 100 μg/mL showed a reduced antitumoral effect, suggesting that LfcinB(20–25)_4_ might induce an antigenic response, as reported in previous studies [42, 43]. Likewise, we have reported that the use of peptide concentrations below the levels employed in the present studies did not have a significant cytotoxic effect in our *in vitro* OSCC model [7]. Based on this, concentrations of LfcinB(20–25)_4_ and LfcinB(20–25) below 100 μg/mL and 300 μg/mL, respectively, were not tested.

To gain insight into OSCC progression due to peptide administration, we examined the histologically features of the tumor at five weeks of treatment. Histological assessment showed that LfcinB(20–25)_4_ peptide induced extensive matrix acellularity in the tumors, an indicator of necrosis caused by a membranous-lytic mechanism [18]. Specifically, the necrotic effect was found only under acute treatment by using a higher concentration of LfcinB(20–25)_4_ (300 μg/mL), while apoptosis was the main mechanism of cell death at lower concentration (100 μg/mL). These findings might be explained by the different peptide concentrations. Low concentrations cause mild damage to the membrane, inducing changes in the homeostasis such as imbalance of the ionic gradients across the plasma membrane, triggering an apoptotic process [44–47]. Conversely, high concentrations could produce the complete disruption of the membrane, leading to necrotic cell death [23, 24]. These data are consistent with previously reported data which show that the peptide concentration as well as the type of treatment (chronic or acute) can lead to different types of cell death (necrosis or apoptosis) [48].

In addition to histological events, we detected severe leukocyte infiltration (lymphocytes, plasma cells, eosinophils, macrophages, neutrophils, among others) in tumors, which was proportional to the dose of the delivered peptide. A recent study reported that cell lysis promoted by LfcinB25-derived CAPs, causes leukocyte infiltration through the release of damage-associated molecular pattern molecules (DAMPs), such as high-mobility group box protein 1 (HMGB1) or adenosine triphosphate (ATP) [18]. Our data suggest that severe leukocyte infiltration could be the cause of the decrease in the antitumoral effect of the LfcinB (20–25)_4_ peptide at higher concentrations_._ Indeed, several studies have shown that dendrimeric peptides can induce an antigenic response [42, 43]. In this context, the present study indicates that higher concentrations of LfcinB (20–25)_4_ drive the activation of specific antibodies, which might cause its degradation.

We next investigated the effect of LfcinB(20–25)_4_ on OSCC CAL27 and non-tumorigenic Het-1A cell structural damage. The *in vitro* results demonstrated that the LfcinB (20–25)_4_ peptide with a cationic charge of +12 provokes an intracellular selective, rapid and disruptive effect on the cell membrane of CAL27 cells after 15 min of treatment. In contrast, LfcinB(20–25) with a cationic charge of +3 does not initiate a severe cell membrane damage in these cells, but produces a significant increase in the intracellular vacuole formation after 45 min. These data suggest that persistent cell stress leads to cell death [49, 50]. In particular, we found that LfcinB(20–25) cytotoxic effect was of 45% in CAL27 cells after 24 h of treatment [7].

Cumulatively, our results indicate that the difference in effectiveness between the LfcinB(20–25)_4_ and LfcinB(20–25) peptides might be linked to both the increase in the cationic charge and the hydrophobicity of LfcinB(20–25)_4_ (i.e. enhanced amphipathic characteristics). This outcome is supported by the study conducted by Hoskin et al. They demonstrated that the relevance of the cationic charge is due to the addition of seven arginines to LfcinB (20–25) (charge +10), which gives rise to a highly cytotoxic and selective peptide in leukemia and lymphoma cells. Furthermore, this modification significantly enhances the cytotoxicity effect of the LfcinB25 peptide [15, 25]. On the other hand, Gifford et al. explained that the peptides need at least a net positive charge of +7 in order to exhibit antitumoral activity [16], an observation that is consistent with our results. In addition, several authors have described that the cationic charge in CAPs plays an essential role in selective cytotoxicity [10, 17, 18, 51] since many cancer cells overexpress anionic molecules on their surfaces (such as phosphatidylserine, O-glycosylated mucins, sialic acid and heparan sulfate [23]). This negative charge will allow strong electrostatic interactions to form between the CAPs and the cell surface, leading to selective peptide binding [23, 52].

Cationic CAP charge and hydrophobic amino acids are both important in determining peptide selectivity. Once the peptide-membrane interaction has been achieved, the hydrophobic amino acids penetrate the lipid bilayer, provoking the destabilization and disruption of the cell membrane. This damage is dependent on peptide concentration [19, 23, 53–55]. Therefore, increasing the hydrophobicity of LfcinB25-derived peptides improves their cytotoxic activity, as shown for LfcinB(20–25)_4_ in our study. Rekdal et al. revealed that substitution of tryptophan in LfcinB25-derived peptides with an artificial aromatic amino acid with higher hydrophobicity, such as 2,4,6-Tri-tert-butyl-N-methylaniline (Tbt), improved the selective cytotoxicity of the peptides in leukemia, rhabdomyosarcoma, and colorectal adenocarcinoma cells [14].

By comparing our *in vitro* and *in vivo* results, we found that the highest concentration of LfcinB(20–25)_4_ induced the most severe cytotoxic effect by activating necrotic cell death. Similarly, Eike *et al*. demonstrated that high concentrations of lactoferricin-derived CAPs caused a necrotic cell morphology with loss of plasma membrane integrity in a human melanoma model. When using low concentrations, the depolarization of mitochondria and release of cytochrome C were induced, which are features indicative of apoptosis [10].

In summary, the results from this study revealed that the tetrameric peptide LfcinB(20–25)_4_ could be considered as a new therapeutic agent for the treatment of OSCC, due to its selective antitumoral effect at low doses. Furthermore, it could be an excellent candidate to use in conjunction with polyethylene glycol, acyl chains, N-(2-hydroxypropyl) methacrylamide and elastin-like polypeptides, macromolecules that could provide improved stability and more controlled delivery with limited immunogenic effect.

## Supporting information

S1 Fig Follow-up antitumoral effect of LfcinB peptides in OSCC hamster model. Tumor macroscopic characteristics were evaluated after five weeks of peptide or vehicle administration. Representative images of tumor size of the buccal pouch for different doses. Representative data of n = 7 animals for vehicle group and n = 5 animals for others groups.
(TIF)

S2 Fig *In vitro* cytotoxic effect of LfcinB (20–25)_4_ on CAL27 cell line. Structural damage caused by LfcinB (20–25)_4_ was assessed via transmission electron microscope. Images show sections of CAL27 pre- and post- treatments.
(TIF)

S3 Fig *In vitro* cytotoxic effect of LfcinB (20–25)_4_ HET-1A cell line. Structural damage caused by LfcinB (20–25)_4_ was assessed via transmission electron microscope. Images show sections of HET-1A cells pre- and post-treatments.
(TIF)
