# Supplementary figures and images for: A tetrameric peptide derived from bovine lactoferricin as a potential therapeutic tool for oral squamous cell carcinoma: A preclinical model
Source: PLoS One. 2017 Mar 30;12(3):e0174707. doi: 10.1371/journal.pone.0174707 (PMC5373611; doi:10.1371/journal.pone.0174707)

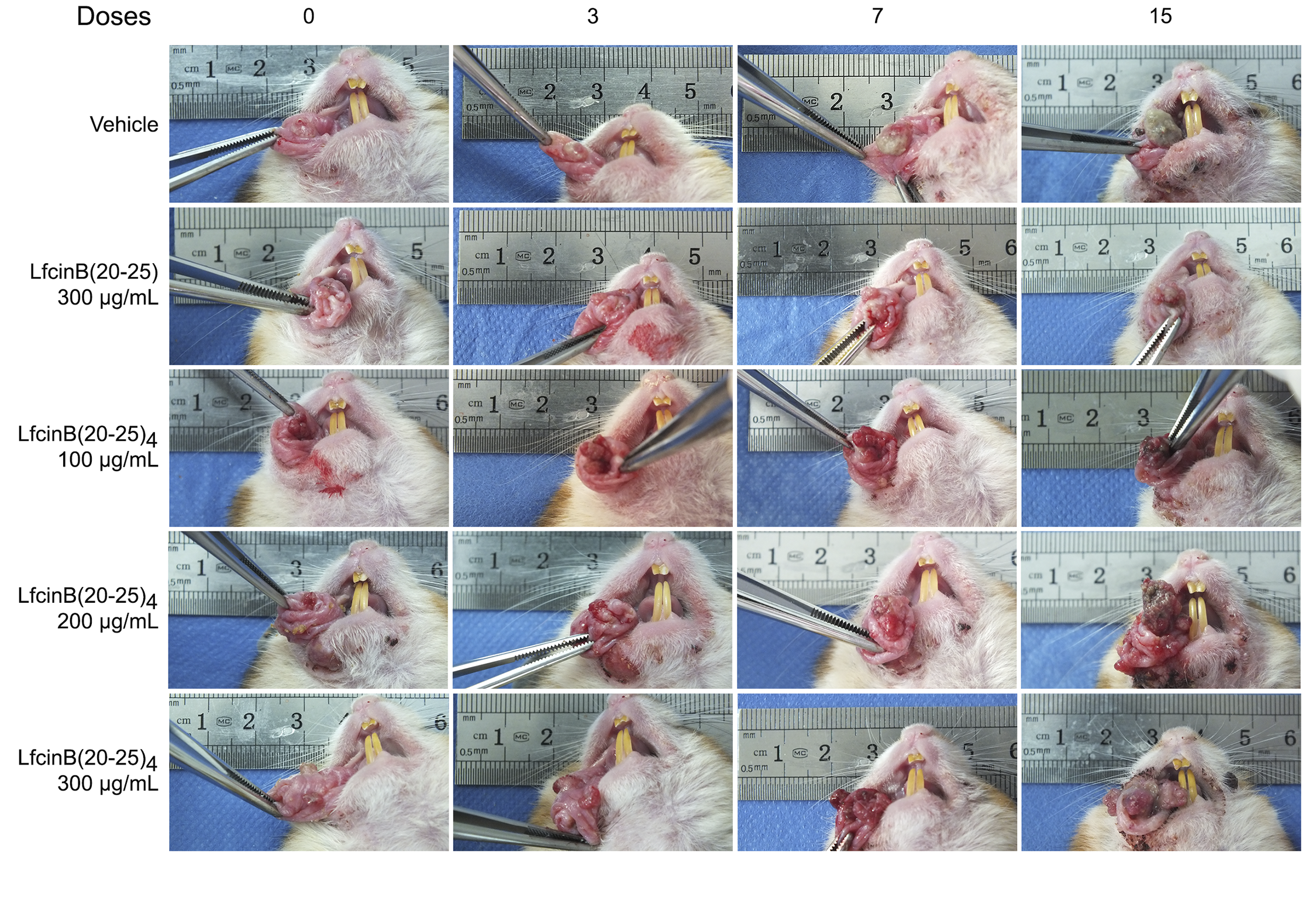

Supplement: S1 Fig — Tumor macroscopic characteristics were evaluated after five weeks of peptide or vehicle administration. Representative images of tumor size of the buccal pouch for different doses. Representative data of n = 7 animals for vehicle group and n = 5 animals for others groups. (TIF) [file pone.0174707.s001.tif]

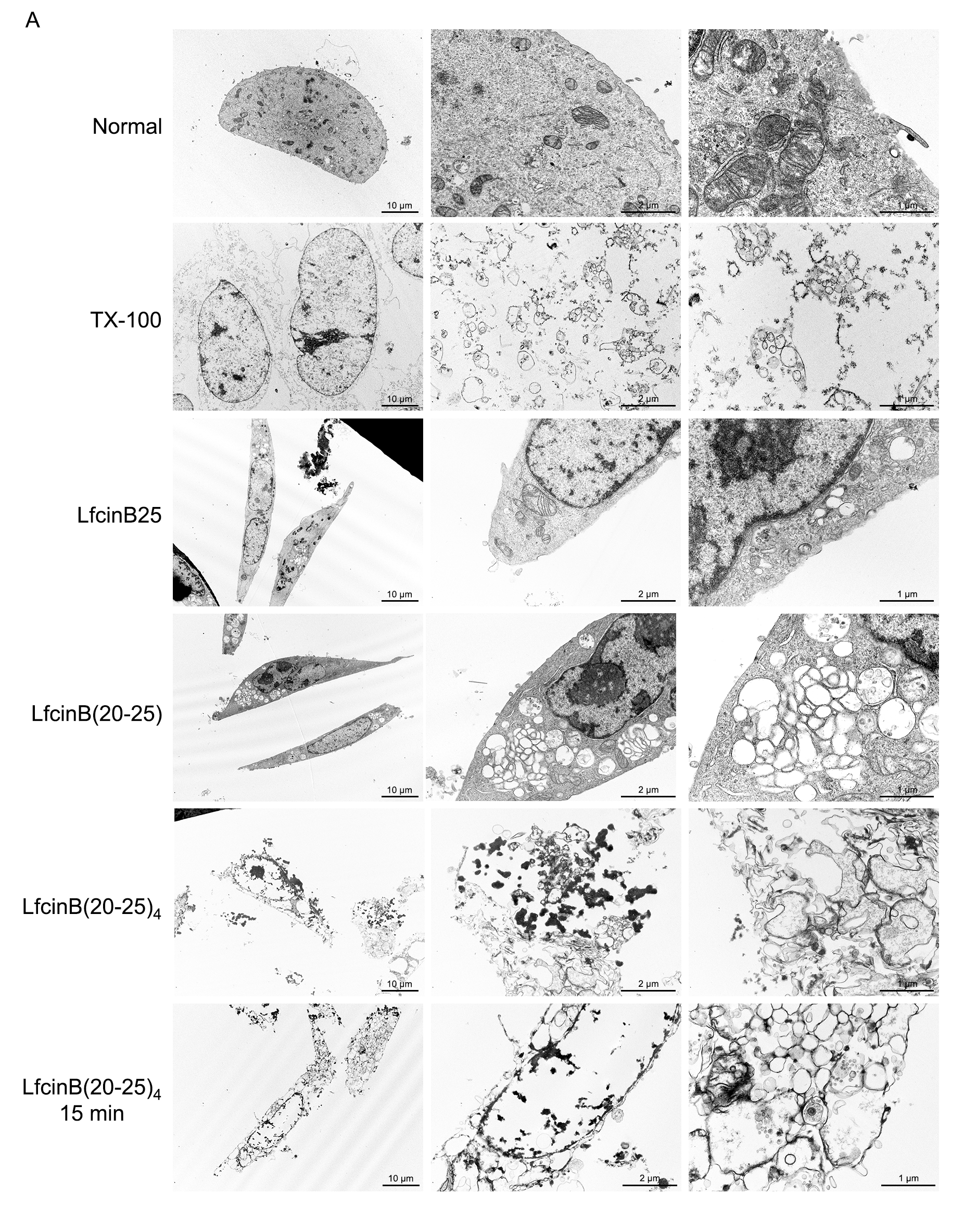

Supplement: S2 Fig — Structural damage caused by LfcinB (20–25)4 was assessed via transmission electron microscope. Images show sections of CAL27 pre- and post- treatments. (TIF) [file pone.0174707.s002.tif]

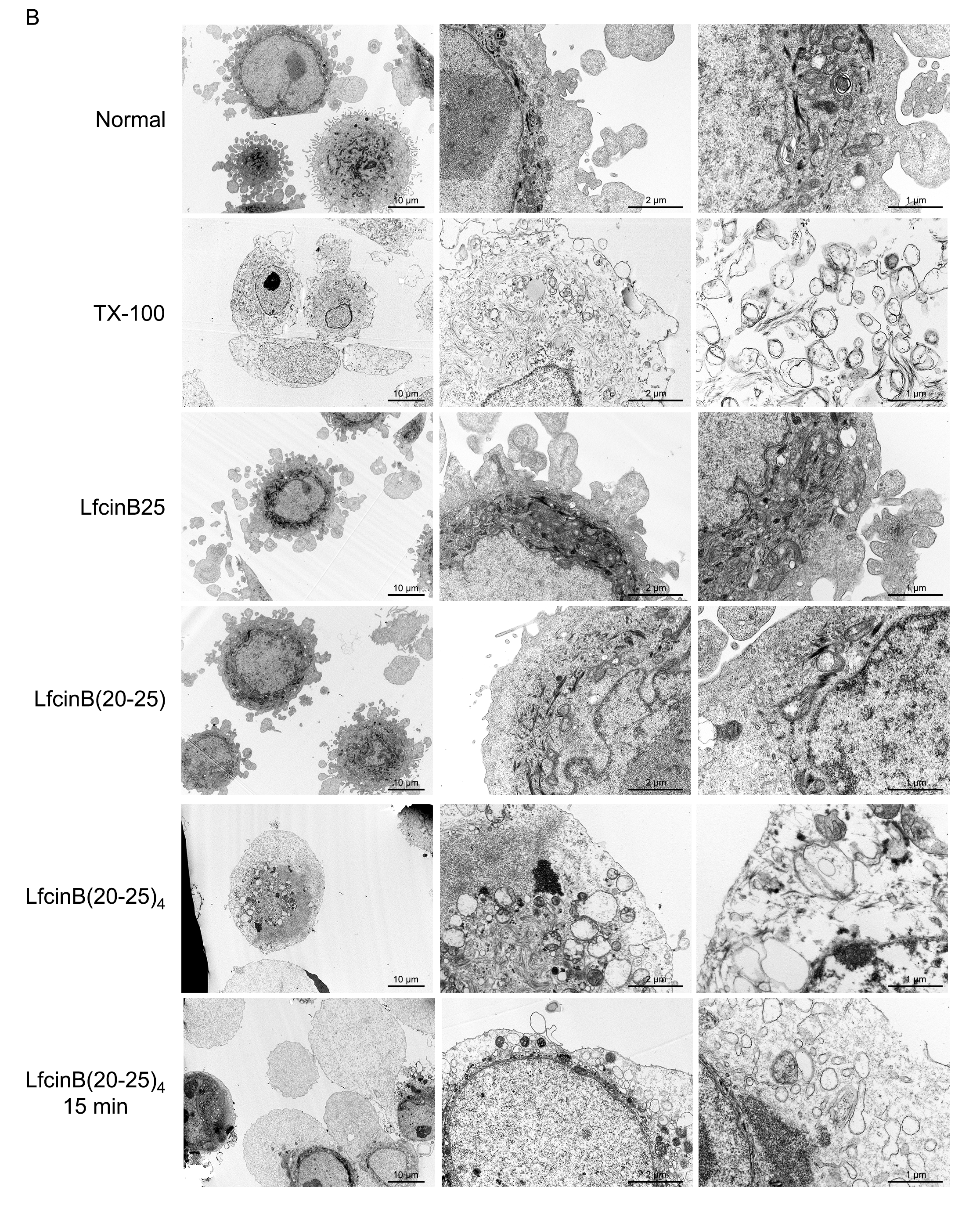

Supplement: S3 Fig — Structural damage caused by LfcinB (20–25)4 was assessed via transmission electron microscope. Images show sections of HET-1A cells pre- and post-treatments. (TIF) [file pone.0174707.s003.tif]
